# Beam and sample movement compensation for robust spectro-microscopy measurements on a hard X-ray nanoprobe

**DOI:** 10.1107/S1600577521007736

**Published:** 2021-08-19

**Authors:** Paul D. Quinn, Miguel Gomez-Gonzalez, Fernando Cacho-Nerin, Julia E. Parker

**Affiliations:** a Diamond Light Source, Harwell Science and Innovation Campus, Didcot, Oxfordshire OX11 0DE, United Kingdom

**Keywords:** feedback, beam positioning, image alignment, nano-XANES, spectro-microscopy, nano­probes

## Abstract

Static and *in situ* nanoscale spectro-microscopy is now routinely performed on the Hard X-ray Nanoprobe beamline at Diamond and the solutions implemented to provide robust energy scanning and experimental operation are described. A software-based scheme for active feedback stabilization of X-ray beam position and monochromatic beam flux across the operating energy range of the beamline is reported, consisting of two linked feedback loops using extremum seeking and position control. Multimodal registration methods have been implemented for active compensation of drift during an experiment to compensate for sample movement during *in situ* experiments or from beam-induced effects.

## Abstract 2

Beam stability and sample drift compensation schemes are presented for use on I14, the Hard X-ray Nanoprobe at Diamond Light Source.

## 1. Introduction   

The emergence of ultra-brilliant synchrotron sources (Tavares *et al.*, 2014 ▸; Liu *et al.*, 2014 ▸), developments in instrumentation (Holler *et al.*, 2018 ▸; Schroer *et al.*, 2017 ▸; Deng *et al.*, 2019 ▸; Nazaretski *et al.*, 2014 ▸) and rapid advances in hard X-ray focusing optics using either reflective (Yamauchi *et al.*, 2011 ▸), refractive (Seiboth *et al.*, 2017 ▸; Patommel *et al.*, 2017 ▸) or diffractive optics (Huang *et al.*, 2013 ▸; Suzuki *et al.*, 2010 ▸; Mimura *et al.*, 2010 ▸) have allowed hard X-ray scanning-probe experiments to offer new possibilities for high-resolution imaging. This potential for new science has resulted in the construction of a number of hard X-ray nanoprobes in recent years, together with planned showcase constructions for new low-emittance facilities (Chang *et al.*, 2013 ▸; Suzuki *et al.*, 2013 ▸; Nazaretski *et al.*, 2017 ▸; Winarski *et al.*, 2012 ▸; Martínez-Criado *et al.*, 2016 ▸; Chen *et al.*, 2014 ▸; Somogyi *et al.*, 2015 ▸; Schroer *et al.*, 2010 ▸; Johansson *et al.*, 2013 ▸; Tolentino *et al.*, 2017 ▸).

Hard X-ray nano-spectromicroscopy is an example of a novel technique which is finding a growing number of applications in a range of scientific areas, with the potential to impact many more.

Delivering nanoscale spectromicroscopy requires robust implementations and solutions to be developed to provide repeatable energy changes, and stable beam positioning and beam intensity during energy scanning. Nano-focusing has additional challenges related to positional stability and any unwanted sample drift over the course of a measurement, particularly during *in situ* experiments.

The I14 Nanoprobe beamline (Quinn *et al.*, 2021 ▸) at Diamond Light Source is a 185 m long instrument designed to enable flexible multi-edge spectromicroscopy on static and *in situ* samples across the 5 to 23 keV energy range of the beamline with a beam size of 50 nm (at 12 keV). Like similar facilities (Nazaretski *et al.*, 2017 ▸; Chang *et al.*, 2013 ▸; Johansson *et al.*, 2013 ▸; Somogyi *et al.*, 2015 ▸) the beamline uses horizontally deflecting optics for all beam-conditioning elements prior to nano-focusing to improve beam stability.

**Figure 1:**
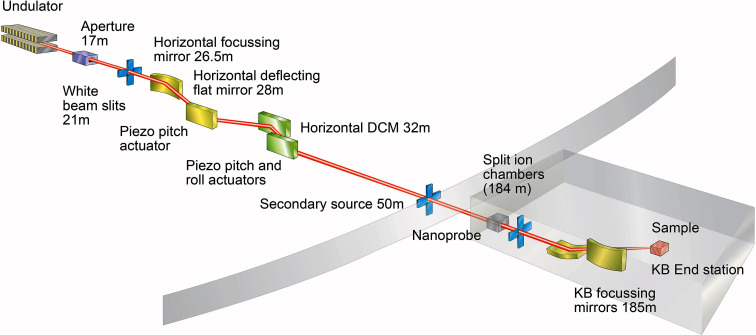
Outline schematic of the nanoprobe beamline (I14) at Diamond Light Source. The beamline uses all horizontal optics for beam conditioning prior to the endstation KB mirrors at 185 m. The pitch and roll piezo actuator and ion chamber measurement positions for beam position and intensity control are indicated.

This horizontal optical arrangement, shown in Fig. 1 ▸, assists with managing vibrations with respect to beam coherence and focusing, but it can present challenges for long-term stability and operation as it results in the coupling of multiple optical elements to the horizontal beam position.

Indirect cooling schemes for the mirrors and the second monochromator crystals can provide low vibrations, but can also result in a slow response to changes in thermal load, which happens when switching mirror stripe or when changing photon energy. The net effect is the slow thermal drift of these elements, happening over several hours in the case of the mirrors. Additionally, Compton scattering from the first monochromator crystal produces a slow temperature increase in the surrounding mechanical elements, resulting in similarly slow drift. Ideally, the metrology design and temperature regulation of the mirrors and monochromator mechanics would compensate for or mitigate these effects, but in practice meeting the required performance can require corrective measures. Mechanical intervention to correct such issues has a major impact on operations and corrective feedback schemes can provide an alternative solution. In this paper we outline the schemes put in place on the I14 Nanoprobe beamline to enable flexible multi-edge spectromicroscopy on static and *in situ* samples.

## 2. Optimization of beam intensity and position   

Feedback schemes using either position- or intensity-based control are typically used to control beamline performance. Intensity-based control requires maximizing the signal, stabilizing the signal at a constant level or fixing the ratio between intensities, such as before and after the monochromator (Krolzig *et al.*, 1984 ▸; Siddons, 1998 ▸). Extremum-seeking control (Krstić & Wang, 2000 ▸; Proux *et al.*, 2006 ▸) uses a ‘perturb and observe’ scheme in which the mechanism controlling the intensity is continuously perturbed and the variation in intensity is measured to determine the location of the maximum and/or direction to move towards the maximum position. The perturb and observe scheme has been implemented in hardware in a number of different ways, but at its core a sine-wave oscillation is used to drive the intensity perturbation by oscillating a monochromator crystal, and a phase-lock loop or phase measurement observes the intensity variation to determine the direction and magnitude (Zohar *et al.*, 2016 ▸; Bloomer *et al.*, 2013 ▸; Stoupin *et al.*, 2010 ▸; Renevier *et al.*, 2003 ▸). Beam position can also be used as an indicator of monochromator alignment and is controlled using piezo actuators in the monochromator. If the source of intensity loss or beam movement is misalignment of the second crystal with respect to the first, a position-based feedback will bring the beam back to the same position, and consequently the monochromator crystals into a parallel alignment (Ramanathan *et al.*, 1988 ▸).

Implementations of intensity-based schemes, while successful, are often localized to a beamline or facility, or implemented for specific hardware, which can hamper more general adoption. Schemes developed at Diamond (Bloomer *et al.*, 2013 ▸), for example, were not straightforward to implement on different beamlines due to differences in control system architectures (*e.g.* older VME versus newer Ethercat). The traditional phase-lock analogue hardware schemes can also be more complex to implement on a beamline with very long distances between sensors, phase loops and actuators. A key factor for nanoscale measurements is also the amplitude and frequency of the oscillation, which must have a negligible effect on the measurement. Implementations of oscillation and phase-lock schemes can require adapting the amplitude of the oscillation with energy as the sensitivity changes with the Darwin width, and these schemes have largely been deployed at facilities operating mainly at fixed energy for diffraction measurements rather than spectroscopy. Beam-position control is more straightforward, with traditional proportional–integral–derivative control loops (PID) used to actuate and control to a fixed setpoint.

In an all-horizontal geometry, several components can influence the horizontal position and intensity. Drift can occur in the pitch of the mirrors before the monochromator which will result in positional and angular change. With the mirror before the double-crystal monochromator (DCM) this angular drift can potentially affect the energy calibration of the monochromator, particularly at higher energies or lower angles, and result in a reduction in intensity if moved sufficiently off the rocking-curve peak. A drift in the angle of the second crystal with respect to the first crystal will also result in a positional change and drop in intensity. A single PID control scheme actuating the monochromator to keep a fixed beam position will not keep the crystals parallel as it would also compensate for mirror drift and result in both intensity loss and possible variation in the energy calibration, particularly at high energies where small angular changes have a larger impact.

A software-based feedback system was therefore developed to implement extremum-seeking control of intensity and PID control of the beam position. Both variables are monitored before the optical aperture is defined for the Kirkpatrick–Baez (KB) mirrors, using two ion chambers. The ion chambers feature split electrodes to provide a position measurement and are arranged orthogonal to each other to provide an *X* and *Y* position, respectively (Sato, 2001 ▸). In addition, the chambers are fitted with an additional grid between the anode and cathode to suppress the effect of slow-moving ions and improve the temporal response (Müller *et al.*, 2013 ▸). The ion chambers were preferred over a single-crystal beam-position monitor (BPM) to avoid beam artefacts from the electrode patterning.

For the intensity tracking the second crystal of the DCM is swept ± 100 nrad in 25 nrad steps at a step rate of 5 Hz through *EPICS* (*Experimental Physics and Industrial Control System*; https://epics-controls.org/epics). The scheme uses a simple hill-climbing type approach such that, when a maximum is found, the centre of the oscillation is moved to that position. This angular modulation is much smaller than the Darwin width of the crystals. In the worst case for this system, using Si(111) crystals at 25 keV, the smallest Darwin width corresponds to 10 µrad. A variation of 0.14σ would cause a 1% intensity variation and this modulation is an order of magnitude below this. The fractional contribution propagates to the secondary source aperture (SSA). For positional control, a standard PID loop controls the angular positioning of the M2 mirror.

Two control loops which both influence the same variable, namely position, and in this case beam position, will generally not work well together if they are not aware of each other in some way. In a typical fully characterized control system with two control loops, the response of the system to different actuations is characterized, and if actuation in one loop influences another loop the influence is estimated and a corrective term is applied, and vice versa to stop the control loop responding unnecessarily. However, in our case the loop is monitored and updated relatively slowly at 5 Hz compared with the actuation and response time so the temporal lag is negligible. The step size or changes in operation are also relatively small to provide gentle correction and variations during an experiment. Rather than have two independent loops we opted to interlace the control loops, whereby the intensity loop steps in its oscillatory pattern, and in between each oscillation step the PID loop is updated and a corrective step is estimated and taken. An overview of the algorithm is presented in Fig. S1 in the supporting information.

The rate of convergence to the maximum intensity can vary so some settling is needed for larger energy steps. To accommodate this variation when taking energy steps greater than 1 keV, the demanded change is split, via software, into four steps with a 30 s pause between steps to allow for both settling and accurate tracking over the energy change. A long beamline also has a tighter tolerance for the beam-pointing direction. A 10 µrad movement over 150 m would put the beam outside the BPM window so this split also helps to correct and track across larger scale changes. For an energy change greater than 2 keV this results in a delay of up to 120 s before the energy move is considered complete and the beam stabilized and optimal. While this would perhaps be considered a long duration for a bulk EXAFS beamline, this timescale is still small compared with the acquisition time for large X-ray fluorescence (XRF) maps or XANES maps.

**Figure 2:**
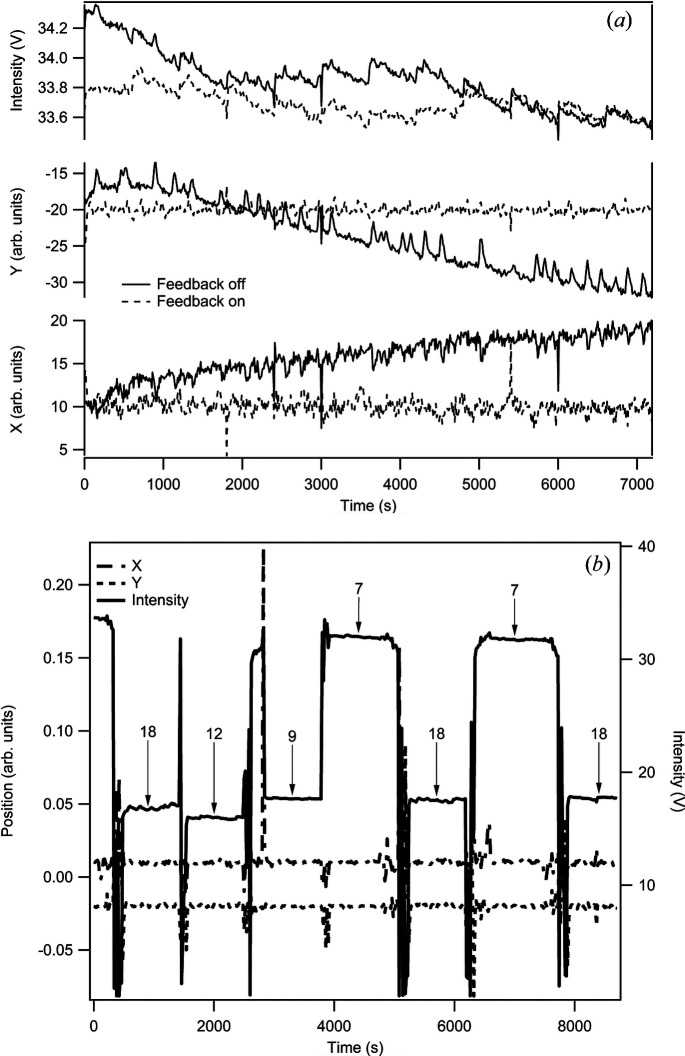
(*a*) Plots of the position and intensity drift observed with and without feedback over a 2 h period. The 10 min top-up cycle is visible in the intensity and position variation. The slow cycles in the positions without feedback operation, particularly in *Y*, are not currently understood but are actively corrected by the feedback. (*b*) Position and intensity as energy is adjusted across a wide range. The perturbations at the beam-position monitor during the energy changes are included. The intensity is displayed in amplified volts for scaling. The ion chamber arrangement consists of two split ion chambers or four amplified signals corresponding to a 0–40 V range.

To demonstrate the operation of the feedback system, measurements of the drift in intensity and beam position with and without feedback were collected [Fig. 2 ▸(*a*)]. The measurements were taken after a large energy move (10 keV) to show the influence of a change in heatload. Fig. 2 ▸(*b*) shows the feedback in operation, plotting the intensity and positions of the beam before the KB mirror for various changes in energy. The feedback is always on during energy moves and operations; it is only paused when a user enters the hutch. The noisy jumps observed between energy moves are partly the motion of the beam as the energy changes, but also due to automatic gain changes on the amplifiers of the beam-position monitors as the flux changes. This scheme has been in operation for two years on the beamline, and it allows for robust and repeatable energy changes across the operating range and reaches maximum intensity in all cases. The energy demands can easily be set by beamline users, and the scheme has allowed a variety of experiments to proceed remotely with minimal staff intervention. Future developments could incorporate a Kalman filter for gradient estimation to accelerate convergence to a peak value.

## 3. Active drift compensation   

Drift of the measured area across multiple measurements is a common problem experienced in X-ray microscopy and can occur for several reasons. *In situ* environments allow investigations under static or flow conditions of liquid or gas, under different pressures or flow rates, which can cause motion of the sample through changes in thermal gradient or flexing of the windows or membranes containing the sample (Fam *et al.*, 2019 ▸; van Ravenhorst *et al.*, 2019 ▸). Nano-focused X-ray beams, depending on the flux and absorption properties, can also lead to localized thermal heating. This localized heating can induce sample movement due to local thermal expansion, with temperature variations that depend on the sample composition and the position of the beam on the sample, resulting in sample movement which will vary depending on the scanning time and any spatial variations in the materials under investigation (Wallander & Wallentin, 2017 ▸).

The relative movement of the beam with respect to the sample can also be an issue. Nano-positioning schemes based on the use of interferometers measure the relative movement of stage and optic to correct for both slow thermal drift and vibrations. These interferometer-based schemes have grown increasingly sophisticated, but to date the authors are only aware of implementations for diffractive or on-axis type focusing optics. Implementations of reflective nano-focusing KB mirrors have controlled the stage position using interferometers and tight temperature control of the room, but interferometric control of the KB relative to the stage has, to date, not been achieved (Villar *et al.*, 2018 ▸; Martínez-Criado *et al.*, 2016 ▸). The measurement of position with a metrology system is never truly a measurement of the actual sample position. It is a measurement of a virtual point or reference point along the direction of the motion-stage movement. Localized movement of the sample with respect to the metrology measurements can still be observed to a varying extent depending on the conditions of the experiment.

The impact of this drift on the experiment is often to force the experimenter to expand the field of view of the measurement to include empty areas such that across the measurements of the experiment the area of interest does not drift out of the field of view and so can be subsequently aligned and analysed (Zhao *et al.*, 2019 ▸), but this can increase the experimental measurement time significantly and the nature of the drift in both direction and magnitude is unknown. Alternatively, only large areas with some strong visible features, such as edges, are selected to help later registration, which can also limit scientific study across a sample.

To minimize the impact of drift an active compensation scheme was implemented which uses active image registration to estimate and correct for the movement. The registration scheme needed to allow for multi-modal images, be robust to contrast changes and allow both small and large scan area sizes. A number of approaches exist for image registration, from the sum of the squares of the differences to Fourier-transform-based cross correlation (Guizar-Sicairos *et al.*, 2008 ▸), but these methods do not incorporate the multimodal registration or robustness to contrast change, and early testing showed issues with scan regions with small numbers of pixels.

In medical image registration, a concept from information theory, mutual information (MI), or relative entropy, is extensively used as an image-matching and multimodal image-matching metric. MI measures the statistical dependence between two random variables or the amount of information that one variable contains about the other. It makes no assumption of the functional form or relationship between image intensities in the two images and so there are no constraints on the image content of the modalities involved (Maes *et al.*, 1996 ▸; Luan *et al.*, 2008 ▸; Xie *et al.*, 2003 ▸). The MI metric can be described in terms of the entropy of the variables. Given two random variables *A* and *B*, the entropy measures the degree of uncertainty in the variables or the dispersion of the probability distribution. The mutual information *I*(*A*, *B*) measures the amount by which the uncertainty about *A* decreases when *B* can be formulated as the distance between the joint entropy *H*(*A*, *B*) and the individual entropies *H*(*A*) and *H*(*B*) such that
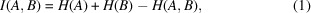
where
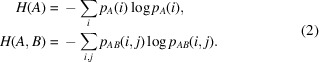



In terms of image registration, alignment of the images *A* and *B* reduces the joint entropy or uncertainty about *B* when *A* is present and vice versa. The estimation of the marginal probability distributions ρ_*A*_ and ρ_*B*_, and the joint probability distribution ρ_*AB*_, is achieved by taking a histogram or joint histogram of the data. The estimated probability distribution will depend on the choice of the bin width. In our implementation, when the number of points *n* is less than 400 the Sturges rule (Scott, 2009 ▸; Sturges, 1926 ▸) is employed to estimate the number of bins, *N*_bins_,
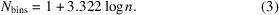
For larger numbers of points the bin width is calculated from the standard deviation of the data set, σ, using Scott’s rule (Scott, 1979 ▸),
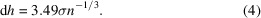
While there are several advanced options for estimating the number of bins to best represent the distribution, we have used the most common empirical estimations, which assume a normal distribution (but apply generally), for ease of use and speed and found them to be robust.

The size of the overlapping part of the images can influence the mutual information metric so a normalized form (NMI) is more widely used and implemented here:
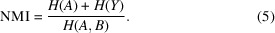



The NMI registration criterion presented here states that the NMI of the image intensity values is maximal if the images are geometrically aligned but limits the metric range to between zero and one. In active drift compensation, the current image is compared with a reference image and the estimated shift between the images is used to adjust the centre of the next scan to compensate for the drift. Optimization methods, while commonly used for image registration, are subject to local minima and the goal was a robust scheme, so a simple 3×3 pixel search grid is used in integer scan steps to find the image shift. During active compensation, registration is only measured to the nearest pixel to keep the definition of the scan region consistent in terms of size and the number of points in a row or column. The process happens in an automated manner throughout the scan and keeps the scan region aligned to within a few pixels (irrespective of the scanning spatial size of the pixel) over the course of a XANES scan. There are no assumptions made on the nature of the relation between the image intensities, so this approach allows for registration to occur between different measurements of the same region. This is needed for situations where no other XRF reference exists, other than the edge under investigation, but differential phase contrast or ptychography, for example, can provide a reference image.

**Figure 3:**
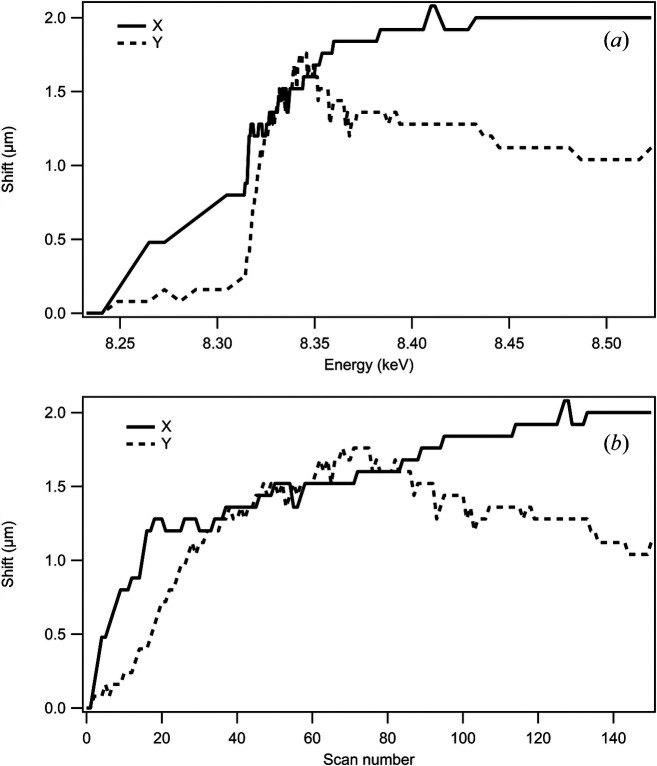
Plots of the sample drift in pixels corrected for in a XANES mapping experiment with active compensation in (*a*) energy (*b*) scan number. Note the correlation of the movement with the Ni absorption edge indicating beam-induced motion.

**Figure 4:**
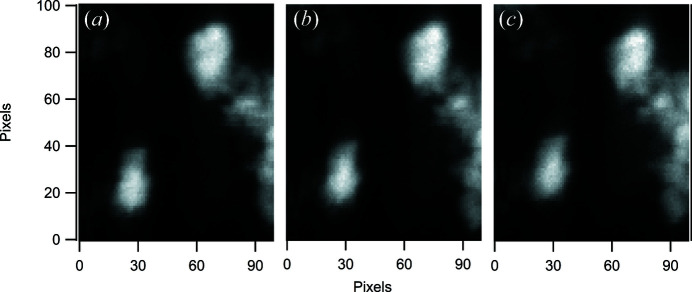
Plots showing Co *K*α XRF maps for a XANES series. The Co *K*α was used as a tracking reference during an Ni *K* XANES map. The plots show (*a*) the first scan, (*b*) the scan at the midpoint of the absorption edge and (*c*) the last scan. The active correction of position maintains the same field of view. The pixel size corresponds to 80 nm.

As an example of the correction of uncontrolled sample movement during an experiment, Fig. 3 ▸ shows the corrections made to a scan of a nickel manganese cobalt battery material during an Ni-edge XANES mapping measurement using the Co *K*α XRF map as a tracking reference. This experiment displayed particularly large sample movement during scanning. The sample movement, in the vertical direction, increases as the energy reaches and scans across the absorption edge, clearly indicating a beam-induced movement. Details of the origins of the movement are beyond the scope of this study but as a more general comment, in our experience, the greatest sample motion during XANES mapping is observed on samples supported on silicon nitride membranes. Very little movement is observed on transmission electron microscopy (TEM) grids or focused ion beam attached samples, so the source of the movement appears to be most likely a thermal effect or a result of static charging. Fig. 4 ▸ shows the image sequence of Co *K*α XRF maps, maintaining the object registration within the defined scan region. The corrections during the scan reach 2 µm and amount to 25% of the field of view but, as can be observed, the tracking correction reduces mis-registration to a few percent. The elemental maps are generated by reading from the data file as the data are acquired and simple summation within a window around the XRF peaks of interest or, in the case of differential phase contrast, a masking and centre of mass operation. This provides live feedback to the user and the input for active registration. The registration method needs to be simple, robust and quick, and, while improved registration can be achieved post experiment, this coarse grid search is sufficient to keep mis-registration to an acceptable level. The time required for this active registration is currently impacted by the acquisition and processing scheme used, which results in a lag at the end of acquisition before data are available from disk to perform registration. This currently adds up to 10 s overhead on average per energy step. The active tracking is optional but over two years of operation it has proven essential for robust scanning across different fields of view and sample conditions.

The mutual information registration method is also employed for post-experiment alignment to sub-pixel resolution. While the main application presented here is for XANES, the method has also been used for rocking-curve maps and other scans where drift may occur in an arbitrary manner.

## 4. Conclusions   

We have implemented a robust scheme for energy scanning which controls position and maximizes intensity, allowing both staff and beamline users to change energy easily, without intervention, across a 4.5–23 keV range whilst maintaining nano-focusing at the sample and optimal intensity. To tackle sample drift, an active drift compensation method was implemented which maintains alignment between successive scans. This allows for *in situ* measurements and compensates for unwanted drift, enabling experiments under a range of conditions, and is tolerant to noise and to changes in field of view and contrast. The active correction method also reduces misalignment corrections post experiment. The combination of the two provides a robust platform for hard X-ray spectro-microscopy and *in situ* experiments.

## Supplementary Material

Flow chart of control loop. DOI: 10.1107/S1600577521007736/mo5243sup1.pdf
