# Supplementary material for: Beam and sample movement compensation for robust spectro-microscopy measurements on a hard X-ray nanoprobe
Source: J Synchrotron Radiat. 2021 Aug 19;28(Pt 5):1528–34. doi: 10.1107/S1600577521007736 (PMC8415335; doi:10.1107/S1600577521007736)
Supplement: Supplementary file 1 [file s-28-01528-sup1.pdf]

JOURNAL OF SYNCHROTRON RADIATION

Volume 28 (2021)

Supporting information for article:

Beam and sample movement compensation for robust spectro-microscopy measurements on a hard X-ray nanoprobe

Paul D. Quinn, Miguel Gomez-Gonzalez, Fernando Cacho-Nerin and Julia E. Parker

**Control Loop**
Initialise $DCM_{pitch}, DCM_{roll}, Mirror_{pitch}$<br>Initialise $PID,\ Intensity_{max} = \emptyset$,<br>Set k oscillation steps, $S_k = [s_1, \dots, s_k]$<br>Read X, Y<br>**While True:**<br>**for** $i = 1, \dots, k$ **do**<br>Update PID_x, PID_y parameters from *X, Y*<br>Update $DCM_{roll}, Mirror_{pitch}$<br>$DCM_{pitch} = DCM_{pitch} + s_i$<br>$Read\ X, Y, Intensity$<br>**if** $Intensity_{max} < Intensity$ **then**<br>$s_{max} = s_i$<br>**end if**<br>**end for**<br>$DCM_{pitch} = DCM_{pitch} + s_{max}$
/

Figure S1 Control Loop used for maximizing intensity and setting the X, Y beam position.
